# Finite element analysis of biomechanical effects of mineralized collagen modified bone cement on adjacent vertebral body after vertebroplasty

**DOI:** 10.3389/fbioe.2023.1166840

**Published:** 2023-07-06

**Authors:** Cunheng Yang, Fumin Wang, Xingxing Huang, Hao Zhang, Meng Zhang, Junxiao Gao, Shengbo Shi, Fuyang Wang, Fangjun Yang, Xiaobing Yu

**Affiliations:** Affiliated Zhongshan Hospital of Dalian University, Dalian, China

**Keywords:** vertebroplasty, finite element analysis, osteoporotic vertebral compression fractures, bone cement, mineralized collagen

## Abstract

**Objective:** To investigate whether mineralized collagen modified polymethyl methacrylate (MC-PMMA) bone cement impacts the implanted vertebral body and adjacent segments and the feasibility of biomechanical properties compared with common bone cement in the treatment of osteoporotic vertebral compression fractures (OVCF).

**Methods:** A healthy volunteer was selected to perform a three-dimensional reconstruction of the T11-L1 vertebral body to establish the corresponding finite element model of the spine, and the changes in the stress distribution of different types of cement were biomechanically analyzed in groups by applying quantitative loads.

**Results:** The stress distribution of the T11-L1 vertebral body was similar between the two bone types of cement under various stress conditions.

**Conclusion:** Mineralized collagen modified bone cement had the advantages of promoting bone regeneration, good biocompatibility, good transformability, and coupling, and had support strength not inferior to common PMMA bone cement, indicating it has good development prospects and potential.

## 1 Introduction

With the development of medical technology, the human life span is also increasing, which will also lead to the population ageing aggravated and then cause the increasing incidence of osteoporosis and vertebral compression fractures (Zhu et al., 2020), a serious problem threatening human health. Percutaneous vertebroplasty was initially used to treat hemangioma, then to treat osteoporotic vertebral compression fractures, with remarkable results (Zhang et al., 2018). Polymethyl methacrylate (PMMA) bone cement, as one of the materials used in vertebroplasty, can provide high strength support (Zhang et al., 2018), reinforces the mechanical strength of the vertebral body (Zhu et al., 2020), and has the advantages of low biological toxicity, easy operation (Charnley, 1960), strong plasticity, high heat release during injection (Lewis, 1997) for rapid analgesia of burned nerve tissue (Yang, et al., 2020a) and restoration of vertebral body height (Zhang et al., 2018). However, PMMA bone cement is also a double-edged sword. Heat released during polymerization will damage the tissues around the affected vertebra, which is not conducive to po stoperative recovery (Gilbert et al., 2000). The high modulus of elasticity of PMMA bone cement is not ergonomic, stresses cannot be uniformly distributed, increasing the chance of injury and adjacent vertebral fractures (Trout et al., 2006). Due to the biological inertness of PMMA bone cement, it does not form meaningful bioincorporation into the injection site (Jiang et al., 2015), resulting in unsatisfactory biocompatibility. Although PMMA cement has been used in clinical practice for almost a century, there remain many defects in PMMA bone cement (Gilbert et al., 2000). Based on the problem of PMMA bone cement, scholars have tried to combine titanium dioxide (Fukuda et al., 2011), alginate, gelatin beads and carboxymethylcellulose (He et al., 2012) with bone cement, However, none of them had satisfactory results.

Currently, with the advantages of mineralized collagen (MC) excellent bone regeneration inducing potential, researchers have tried to combine mineralized collagen with PMMA bone cement, called mineralized collagen modified polymethyl methacrylate bone cement (MC-PMMA). Mineralized collagen modified bone cement has been reported many times in clinical treatment (Zhu et al., 2020), postoperative indicators such as visualanaloguescale (VAS), Oswestry Disability Index (ODI) were significantly better than the common bone cement group, which was quite effective. However, the literature on mineralized collagen modified bone cement is mostly limited to clinical cases and animal experiments, and there is still a huge gap in biomechanical research and understanding its mechanical principles and stress distribution characteristics can help physicians understand and adjust the optimal treatment strategy. The aim of this study was to use the finite element analysis to investigate whether T11-L1 levels behave differently under a variety of stress conditions in the T12 vertebral body using conventional bone cement *versus* mineralized collagen modified bone cement, and whether they provide sufficient support strength to better understand their mechanical principles and properties.

## 2 Materials and methods

**FIGURE 1:**
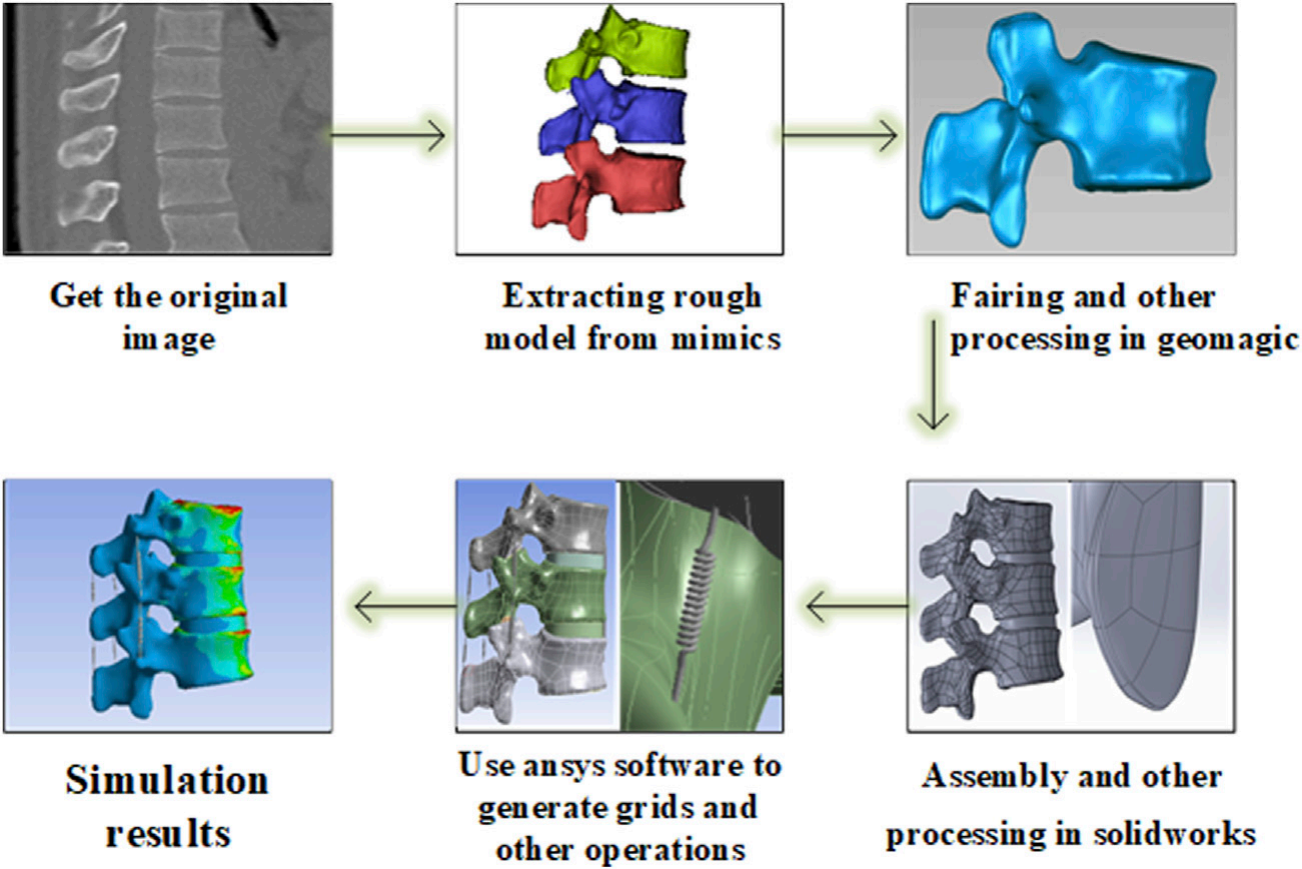
Flow chart of finite element analysis.

First, a three-dimensional finite element model of the three spine segments (T11-L1) was developed using the software from the image data provided by the volunteer. After verifying the validity and feasibility of this model through the previous human *in vitro* experimental data, a simulated object acting as a bone cement was set at the core of the T12 level to simulate the vertebroplasty process. The change of bone cement in the experiment was simulated by changing the assigned value of bone cement, the change of stress was measured, and the biomechanical effect of enhancing the vertebral body and adjacent vertebral body under different cases of bone cement implants was analyzed. According to the numerical changes, it was determined whether there were statistically significant differences in biomechanics between the two, and the flow chart is shown in Figure 1.

**FIGURE 2:**
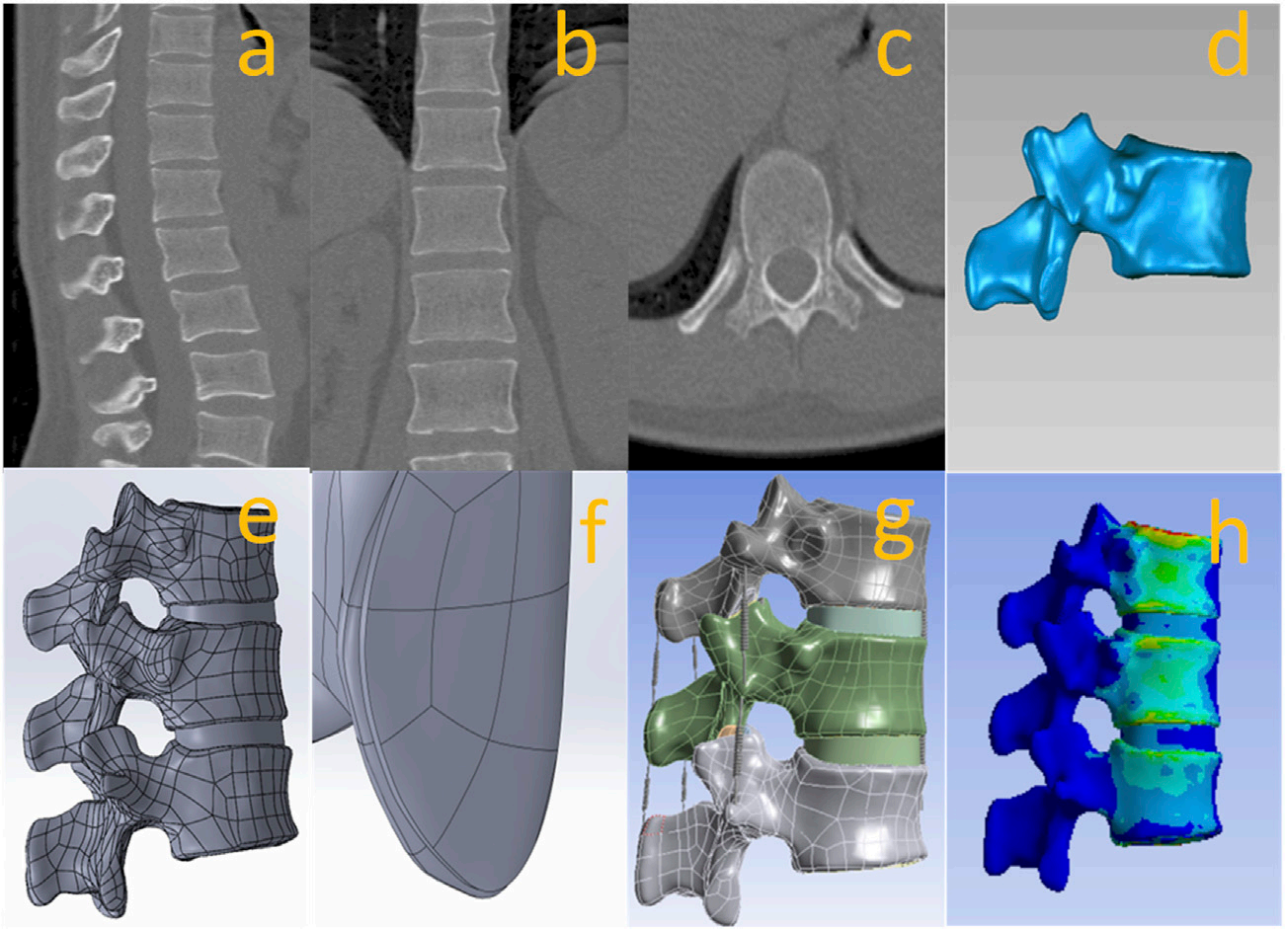
**(A–C)** original image, **(D)** processing in geomagic, **(E)** vertebral body assembly and intervertebral disc reconstruction, **(F)** articular cartilage reconstruction, **(G)** Post-processing such as ligament reconstruction in ansys, **(H)** testing and evaluation results.

We selected a healthy volunteer who did not find abnormalities in imaging data and generated three-dimensional spine geometric models of T11, T12, and L1. The volunteer underwent imaging to obtain information. All CT images were saved in DICOM format and the slice thickness of CT is 1 mm. Image data were imported into the medical 3D reconstruction software MIMICS 21.0, rough models of bone tissue and soft tissue were extracted to construct T11-L1 three-dimensional models, and the vertebral body models were reconstructed from the scanned images and exported to STL format. To generate a more accurate and smoother 3D digital model, the digital geometric model in STL format was imported into Geomagic 2017 software for smoothing and fitting curved surfaces generation and assembly of the vertebral body, intervertebral disc, and nucleus pulposus using software SOLIDWORKS 2021. Finally, the model file was imported into ANSYS 17.0 for mechanical analysis, and the results were obtained. To pursue accurate experimental data, tetrahedral meshing was used for the vertebral body and intervertebral discs. See Figure 2 for more details.

A spring unit was used to simulate the effect of ligaments and joint capsules. The contact relationships between the disc and vertebral bodies, vertebral bodies, ligaments, and between the disc and ligaments were all set as “binding”. The properties of the ligaments were set by stiffness, and the vertebral bodies consisted of 1 mm thick cortical and internal part cancellous bone with 1 mm thick endplates at the superior and inferior edges of the vertebral bodies. We set the cortical shell thickness to 1 mm, while the endplate thickness was also set to 1 mm, articular cartilage was set to 0.3 mm, the disc consisted of nucleus pulposus and outer ring, nucleus pulposus body volume was set to about 40% of the total disc area (Pooni et al., 1986). The above material properties and assigned values (Pintar et al., 1992; Xu et al., 2014; Zhu et al., 2021) are presented in Tables 1, 2.

**TABLE 1:** Ligament stiffness value (unit: N/m).

Anterior longitudinal ligament (ALL)	Posterior longitudinal ligament (PLL)	Ligamentum flava (LF)	Capsular ligament of joint (CL)	Interspinous ligament (ISL)	Supraspinous ligament (SSL)	Transverse ligament (TL)
33	10	24	12	15	32	15

**TABLE 2:** The material properties of the finite element model.

Component name	Modulus of elasticity (MPa)	Poisson’s ratio
Cortical bone	12000	0.3
Loosen cortical bone	8040	0.3
cancellous bone	100	0.3
Loose cancellous bone	33	0.3
Cartilage	10	0.4
Bony endplate	1000	0.4
Nucleus pulposus	1	0.499
Annulus fibrosus	450	0.3
PMMA	1600	0.33
MC-PMMA	1132	0.32

Constant loads were used to simulate loading in this study. Under these non-high-strength loads, the simplified bone material changes linearly with a load. Except for accidents, most fractures are caused by fatigue and accumulation of injuries. However, in this study, the applied loads were transient, and fatigue attributes could be basically ignored. Therefore, it is sufficient to simulate most of the components with an elastic material model.

In the finite element simulation experiment, the mesh is the important factor affecting the simulation accuracy. Generally, smaller mesh sizes may lead to more accurate results, but at a higher computational cost, so the most appropriate mesh size tends to depend on the needs of the research question compared to that. In this study, in order to find the appropriate mesh size, the previous research results were synthesized, and it was decided to use 2.5 mm (Peng et al., 2018) as the optimal size of the mesh for this simulation experiment, which can obtain a comprehensive balance between time and fineness. Validation following the establishment of the finite element model is critical to the accuracy of simulating real feedback using this model. The finite element model was developed using the volunteer data, and the range of motion of the vertebral bodies was calculated and compared with the results of *in vitro* experimental literature to verify the feasibility of the model.

A recent study showed that bone cement injection volume of 6 ml can increase spinal strength (Belkoff et al., 2000), so this study adopted the data of bone cement volume of 6 ml to implant bone cement into the core of T12 vertebral body.

During forward flexion, extension, left and right lateral flexion, and left and right axial rotation movement, an axial force of 300 N was applied downward on the upper surface of the T11 endplate while applying a moment of 7.5 Nm in this direction and restraining the lower end of the L1 segment, and an axial force of 300 N was applied during axial compression and restrain the lower end of the L1 segment.

The host of this experiment was configured with GeForce RTX 3080 Vulcan OC graphics card, the CPU was Intel core i9 9900k, and the disk was SAMSUNG MZVLW512HMJP-000H1. Data analysis was performed using SPSS 23.0 software, and statistical differences were considered at *p* < 0.05.

## 3 Results

Data were obtained and compared with existing literature (Panjabi et al., 1994) under six loading conditions, including flexion and extension, left and right lateral flexion, left and right axial rotation, the range of motion of the model in flexion and extension, left and right flexion, and left and right axial rotation was 6.8°, 7.3°, 6.1°, 6.8°, 2.7°, and 2.6°, respectively, which was highly similar to the literature. Good matching between the experiment and simulation showed that the finite element model established in this study had high accuracy and verified the effectiveness of this model. This also suggested that our model could be used for subsequent studies on bone cement.

**FIGURE 3:**
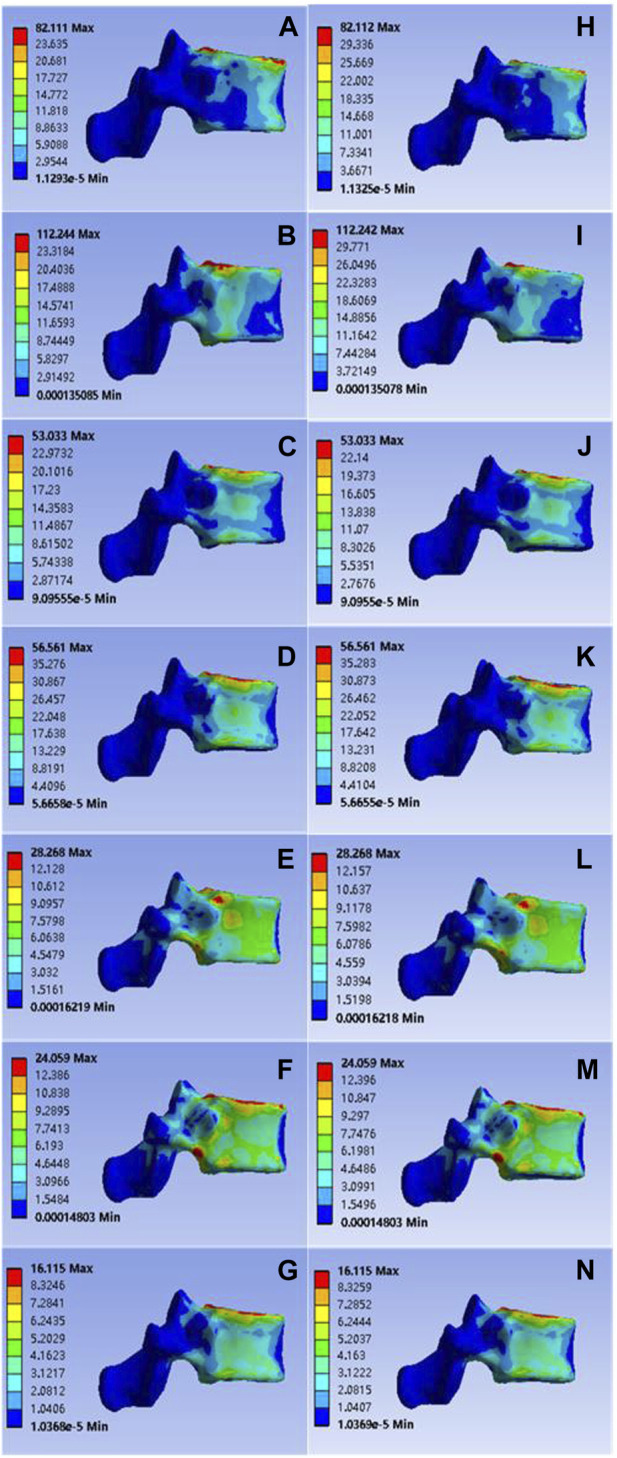
**(A–G)** were respectively the stress distributions of T11 vertebral body subjected to anterior flexion, posterior extension, left-right lateral flexion, left-right axial rotation and vertical compression test when the common PMMA bone cement was implanted, and **(H–M)** and n were the stress distributions of T11 vertebral body subjected to anterior flexion, posterior extension, left-right lateral flexion, left-right axial rotation and vertical compression test when the mineralized collagen modified bone cement PMMA bone cement was implanted.

The calculated data showed that in the case of common bone cement, the values for T11 vertebral flexion and extension, left and right flexion, left and right axial rotation and vertical compression were 82.111, 112.244, 53.033, 56.561, 28.268, 24.059, and 16.115 Mpa, respectively; Corresponding values for the mineralized collagen modified bone cement group were: 82.112, 112.242, 53.033, 56.561, 28.268, 24.059, 16.115 MPa, as shown in Figure 3. Overall, the two groups showed no statistical difference in the presentation of the data values after statistical calculation with SPSS software (*p* > 0.05), indicating that mineralized collagen modified bone cement did not cause an increase in stress distribution at the T11 level.

**FIGURE 4:**
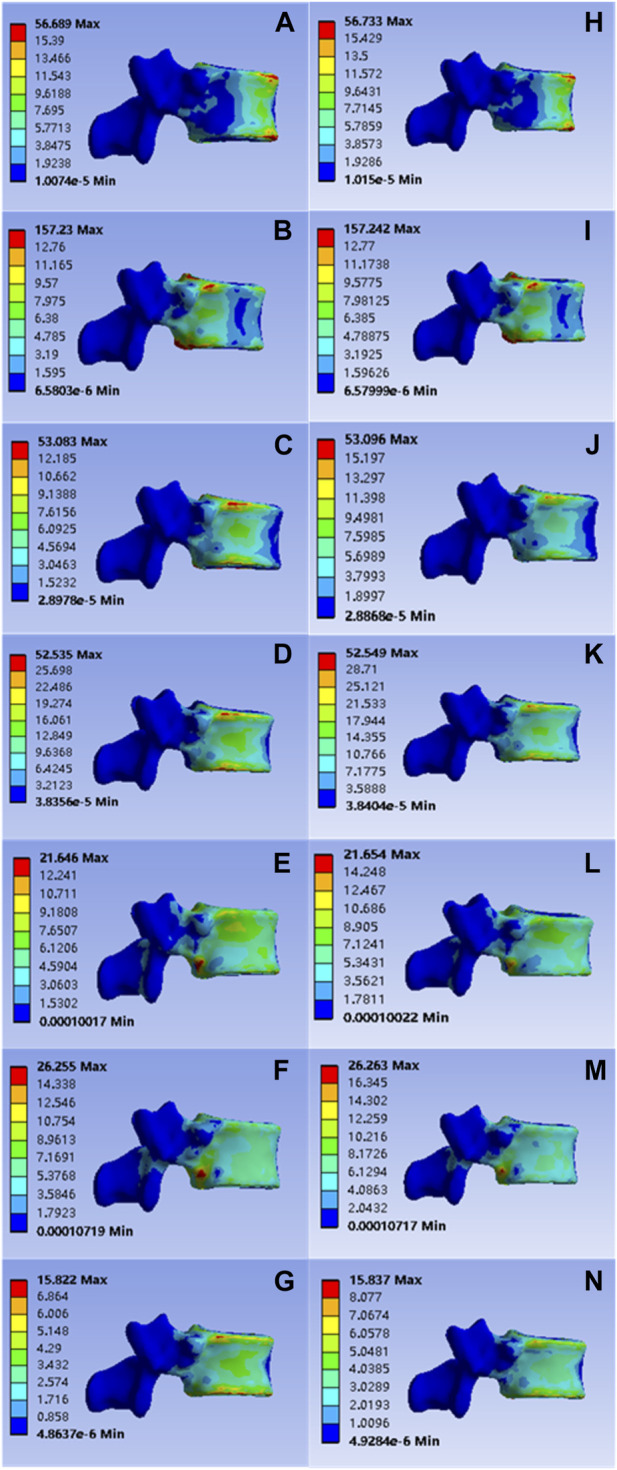
**(A–G)** were respectively the stress distributions of T12 vertebral body subjected to anterior flexion, posterior extension, left-right lateral flexion, left-right axial rotation and vertical compression test when the common PMMA bone cement was implanted, and **(H–N)** were the stress distributions of T12 vertebral body subjected to anterior flexion, posterior extension, left-right lateral flexion, left-right axial rotation and vertical compression test when the mineralized collagen modified bone cement PMMA bone cement was implanted.

After model analysis, the values of T12 vertebral anterior flexion, posterior extension, left-right lateral flexion, left-right axial rotation and vertical compression were 56.689, 157.230, 53.083, 52.535, 21.646, 26.255, and 15.822 Mpa, respectively; Corresponding values for the mineralized collagen modified bone cement group were: 56.733, 157.242, 53.096, 52.549, 21.654, 26.263, and 15.837 Mpa, as shown in Figure 4. The distribution of stress values in the T12 vertebral body of the collagen mineralized modified cement group was statistically calculated to be *p* > 0 .05 compared to the common cement group, which showed no statistical difference and demonstrated that the elastic modulus of the mineralized collagen modified bone cement was reduced within a reasonable range, providing a support strength of this segment that was no less than that of the common bone cement, indicating that the use of mineralized collagen modified cement did not cause a significant increase in the distribution of stress at the T12 level and did not increase the potential risk of fracture.

**FIGURE 5:**
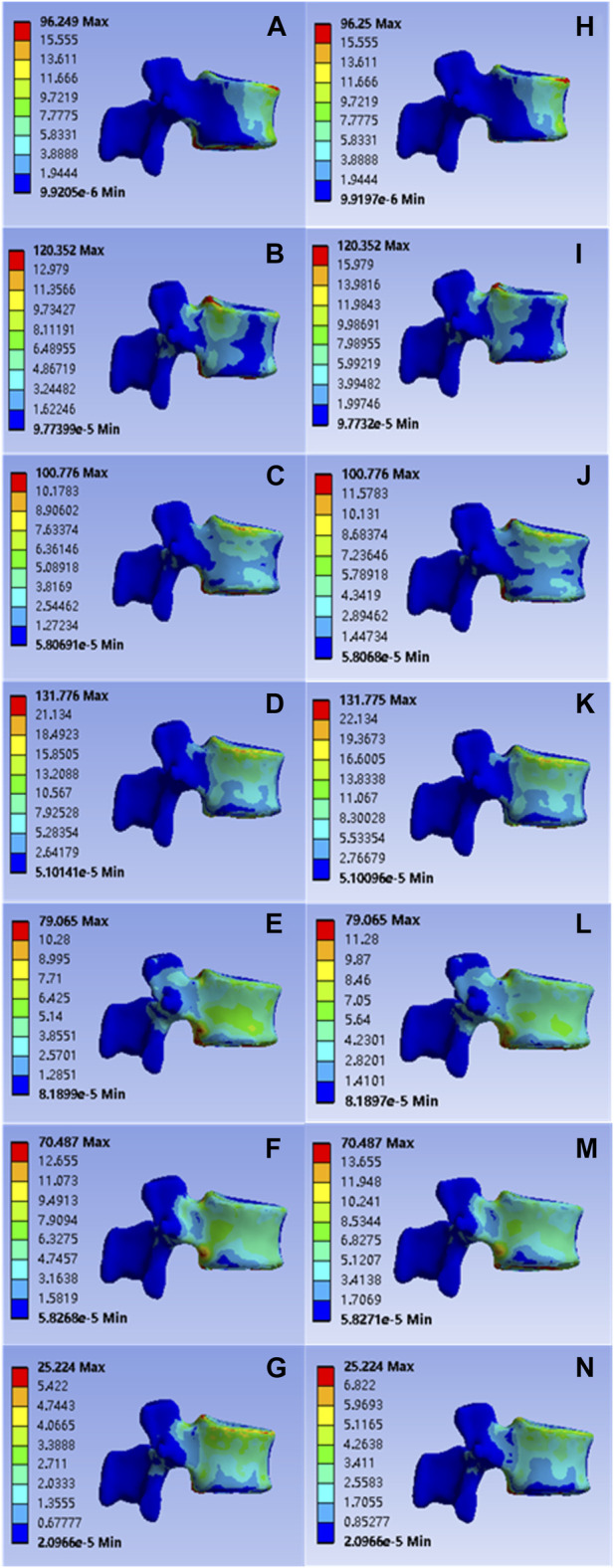
**(A–G)** were respectively the stress distributions of L1 vertebral body subjected to flexion, extension, left and right flexion, left and right axial rotation, and compression test when the common PMMA bone cement was implanted, and **(H–N)** were the stress distributions of L1 vertebral body subjected to anterior flexion, posterior extension, left-right lateral flexion, left-right axial rotation and vertical compression test when the mineralized collagen modified bone cement PMMA bone cement was implanted.

The values of L1 vertebral anterior flexion, posterior extension, left-right lateral flexion, left-right axial rotation and vertical compression of the implanted common bone cement were 96.249, 120.352, 100.776, 131.776, 79.065, 70.487, and 25.224 Mpa; Corresponding values for the mineralized collagen modified bone cement group were: 96.250, 120.352, 100.776, 131.775, 79.065, 70.487, and 25.224 MPa, as shown in Figure 5. Statistical calculations for both groups showed *p* > 0 .05 and no statistical difference, demonstrating a reasonable reduction in elastic modulus for mineralized collagen modified cement, and indicating that mineralized collagen modified cement did not cause an increase in stress distribution at the L1 level.

**FIGURE 6:**
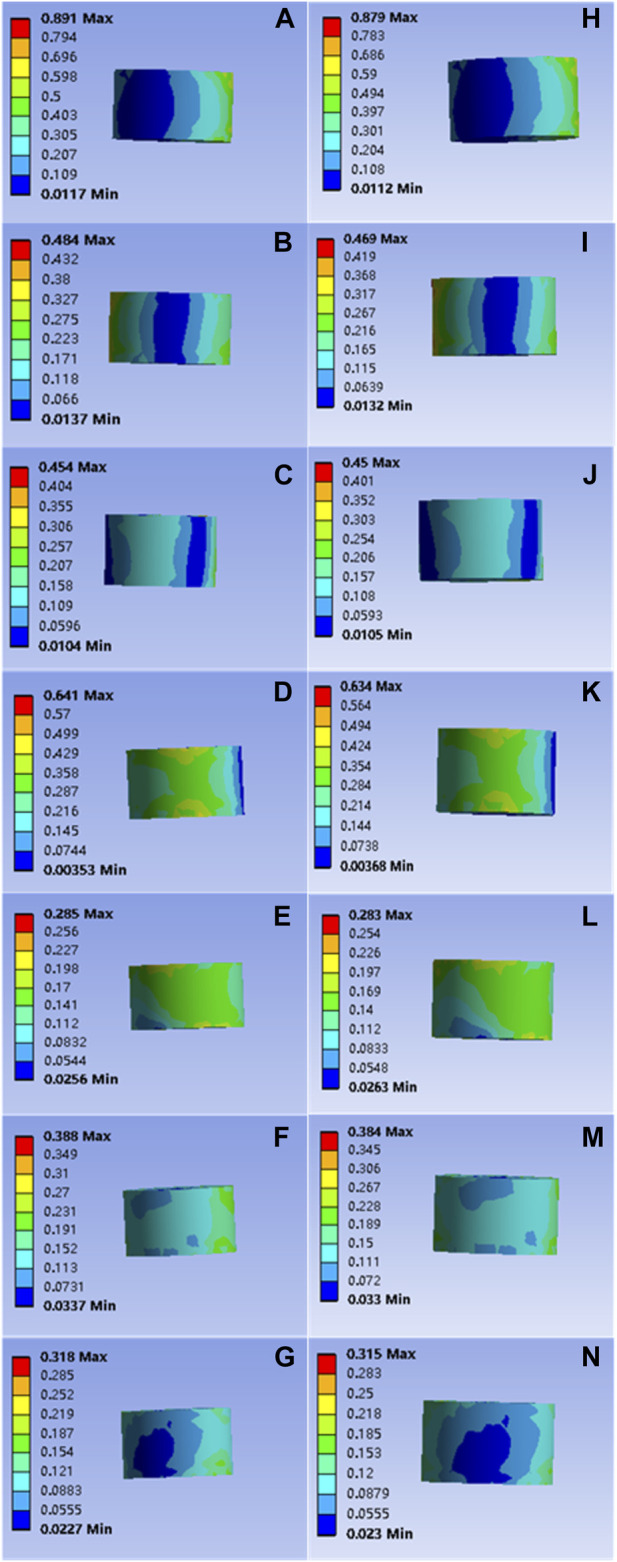
**(A–G)** are respectively the stress distributions of the bone cement subjected to flexion, extension, left and right flexion, left and right axial rotation, and compression tests when the common PMMA bone cement is implanted, and **(H–N)** are the stress distributions of the bone cement subjected to flexion, extension, left and right flexion, left and right axial rotation, and compression tests when the mineralized collagen modified bone cement PMMA bone cement is implanted.

It is important to note that the force applied to the bone cement block was also not significantly compromised. The forces applied to the common bone cement in flexion, extension, left and right flexion, left and right axial rotation, and compression were 0.891, 0.484, 0.454, 0.641, 0.285, 0.388, and 0.318 Mpa, respectively; The forces applied to the modified bone cement block in flexion, extension, left and right lateral flexion, left and right axial rotation, and compression were 0.879, 0.469, 0.450, 0.634, 0.283, and 0.384, 0.315 Mpa, respectively, as shown in Figure 6. Statistical calculation showed *p* > 0.05 between the two groups, and there was no statistically significant difference in stress.

## 4 Discussion

As the elemental composition of layered structure tissue of biological bone tissue, mineralized collagen is an indispensable component in bone (Weiner and Wagner, 1998). Some scholars have found that the incorporation of composite collagen into calcium phosphate bone cement can improve the adhesion of bone cement (Miyamoto et al., 1998), while the use of composite collagen mixed with bone cement can improve the adsorption and tropism of osteoblasts on the surface of calcium phosphate bone cement (Knepper-Nicolai et al., 2002). The use of composite collagen mixed with bone cement has found that the adsorption and tropism of osteoblasts on the surface of calcium phosphate bone cement can be improved. In experiments aimed at the induction of rat bone marrow stem cells with scaffolds composed of layered intra-fibrillar mineralized collagen, it was also found that mineralized collagen scaffolds promoted the adhesion, tropism, proliferation and differentiation of rat bone marrow stem cells in rats, prepared a comfortable microenvironment, and the elastic modulus of newly formed bone in experimental animals was very similar to that of native bone reported (Liu et al., 2016). In addition, there are still many research data to provide evidence to support that MC can promote the repair of bone defects and new bone formation, which shows excellent application prospects and has been used in many fields such as stomatology, orthopedics, and neurosurgery.

In terms of its biosafety, it has been reported that good biosafety has been demonstrated in rabbit joint defect experiments (Li et al., 2015). MC-PMMA bone cement has also been shown to promote healing and osteogenesis at the gene or molecular expression level (Yang et al., 2020b): 1) In sampled macrophages, it was found that the expression levels of genes IGF-1, bFGF and TGF-b and their downstream products were significantly lower than those in the common PMMA bone cement group, while the reduction of these expression products reduced adverse fibrous encapsulation and proliferation (Bank et al., 2017); 2) The MC-PMMA group showed enhanced expression of IL-6 and TNFa genes, which promoted osteoclastogenesis, osteoblastic differentiation of mesenchymal stem cells, and osteoblast migration (Wang et al., 2017), and also showed significantly improved clinical efficacy and follow-up in the case of patients with vertebral compression fractures using MC-PMMA bone cement (Bai et al., 2017; Zhu et al., 2020; Zhu et al., 2021), compared to surgery with common PMMA bone cement.

In addition, mineralized collagen modified bone cement has other significant advantages: 1) the released heat is significantly reduced during cement polymerization, which can retain viable cells to a greater extent and improve the repair effect. 2) The operability of mineralized collagen modified bone cement does not change significantly, and the intraoperative operation time does not prolong significantly (Jiang et al., 2015; Zhu et al., 2020). 3) *In vitro* biomechanical studies by Boger et al. (2007) showed that the strength of failure of functional spinal units could be better maintained using low modulus polymethyl methacrylate.

In recent decades, various methods have been used to modify PMMA bone cement materials, including linoleic acid (LA) (Fagan et al., 2002), chitosan (Herron et al., 2020) and other protocols. However, most of these methods lead to problems because the material does not meet surgical requirements, or the compressive strength is too low. The elastic modulus of mineralized collagen modified bone cement is significantly lower than that of common bone cement (Zhu et al., 2021), but the degree of reduction is within a reasonable range, which can provide the injured vertebra with the necessary strength to maintain vertebral stability, coupled with its characteristics of promoting bone tissue regeneration, strengthen the combination of bone cement and surrounding cancellous bone, reduce the risk of refracture of the adjacent vertebral body of the injured vertebra, and improve the quality of life and prognosis of patients. In this paper, the finite element analysis method is also used to confirm that this scheme is effective at the biomechanical level.

In our current study, the finite element model provided a powerful tool to investigate the biomechanics of compression fracture vertebroplasty. However, some limitations of finite element methods have to be considered. For simplification, the material representation of the biological structure was assumed. Our finite element model was constructed from a CT scan of the normal spine, which was different from patients with compression fractures causing deformation in the spine. Therefore, the simulated loading may differ from patients, which may affect the stress distribution on the spine. It is also important to consider that patients who use mineralized collagen modified bone cement will have bone cement absorbed and dissolved by the human body due to the presence of collagen components, and microcavities may be replaced by bone tissue or form cavities, whether this situation will lead to changes in the internal stress structure and stress situation of the bone cement remains unknown, in addition, the model is not always the same as the clinical case, and there will always be differences in individuals, and the model restriction conditions also include age, sex, race, actual injury status, and so on, which vary from individuals to individuals, and more reasonable conditions and loads can also be set to further assess the situation.

Mineralized collagen modified bone cement provides patients with more suitable fillers for compression fractures due to its good biological coupling and biotransformation, without losing too much support strength and causing sudden local stress change. This simulation experiment provides some theoretical reference basis for the clinical application of collagen mineralized modified bone cement, indicating that mineralized collagen modified bone cement has considerable medical potential and broad space.

## Data Availability

The original contributions presented in the study are included in the article/supplementary material, further inquiries can be directed to the corresponding author.
